# A Retrospective Study on Single-Stage Reconstruction of the Ear following Skin Cancer Excision in Elderly Patients

**DOI:** 10.3390/jcm11030838

**Published:** 2022-02-05

**Authors:** Alberto Bolletta, Luigi Losco, Mirco Pozzi, Michela Schettino, Emanuele Cigna

**Affiliations:** 1 Plastic Surgery and Microsurgery Unit, Department of Translational Research and New Technologies in Medicine and Surgery, University of Pisa, 56126 Pisa, Italy; luigi.losco@gmail.com (L.L.); mircovirgilio.pozzi@gmail.com (M.P.); michelaschettino@gmail.com (M.S.); 2 Plastic Surgery Unit, Department of Medicine, Surgery and Dentistry, University of Salerno, Baronissi, 84081 Salerno, Italy; 3 Department of Plastic and Reconstructive Surgery, Brugmann Hospital Brussels, 1020 Brussels, Belgium

**Keywords:** ear reconstruction, skin cancer of the ear, partial defects of the auricle, single-stage ear reconstruction, local flap, algorithm for ear reconstruction

## Abstract

Ear reconstructive surgery aims to solve the deformities caused by cancer excision. Despite the numerous surgical procedures described, recreating the complex anatomy of the ear still represents a challenge, particularly for young surgeons. The purpose of this exploratory pilot study is to review our experience with single stage reconstruction of the partial defects of the auricle, and propose an algorithm based on defect size, location, and characteristics. We retrospectively reviewed patients who underwent ear reconstruction after cancer excision at our institution between February 2018 and November 2020. The data collected included patients’ demographics, defect characteristics, reconstructive technique used, complications, and outcomes. The patients were evaluated at a minimum follow-up time of 12 months. Forty-six patients were included in the study. The most common cause for ear reconstruction was basal cell carcinoma. The mean area of defect was 4.3 cm^2^ and the helix was the most frequent location of defect. Two patients experienced post-operative complications. At the one-year follow-up, difference in skin pigmentation was reported in 10 cases, a depressed contour of the ear was found in 4 cases, and moderate ear asymmetry was found in 11 cases. No patient needed a secondary procedure. In conclusion, the proposed reconstructive algorithm represents a reconstructive indication that is simple and characterized by low complication rates and good outcomes for both the patient and the surgeon.

## 1. Introduction

The reconstruction of the auricle represents a complex challenge for the plastic surgeon. The different etiologies, the wide range of reconstructive options available, and the patient’s expectations require a global evaluation of the appropriate surgical technique [1]. Moreover, the external ear is an important component of the face, as it guarantees balance between the various facial features and directs the sound waves into the acoustic meatus. It also acts as a support base for glasses, earphones, and hearing aids.

Ear reconstructive surgery aims to resolve the deformities generated by cancer excision, using various techniques capable of restoring the original shape of the auricle. However, some of the surgical procedures described in literature require multiple stages, and this might be an issue for elderly patients [2,3]. 

In the present work, we reviewed our experience with single-stage reconstruction of the partial defects of the auricle after cancer excision. The aim of this exploratory pilot study is to show how principles of single-stage ear reconstruction can be applied to most partial defects of the auricle with satisfying results for both the surgeon and the patient, as it allows for a quick recovery with acceptable outcomes. Moreover, we propose an algorithm based on defect size, location, and characteristics to guide the surgeon approaching this topic.

## 2. Materials and Methods

In this study, we retrospectively reviewed patients treated with ear reconstruction at our institution between February 2018 and November 2020. The inclusion criteria were auricle reconstruction after cancer excision and a minimum follow-up time of one year. The exclusion criteria were smaller defects treated with primary closure, larger defects requiring major ear reconstruction and multiple-stage procedures, patients who did not attend the follow-up, and lack of documentation. The study was performed with respect to the ethical standards of the Declaration of Helsinki, and all patients signed an informed consent form comprising a photo release section. 

The data collected from patients included in the study were patients’ demographics (gender, age, smoke habit, drug assumption, and comorbidities, such as diabetes and cardiovascular disease), histological diagnosis and defect characteristics (size, location, and structures involved), reconstructive technique used, and cancer recurrence and complications. Photographic documentation was also collected. At a minimum follow-up time of one year, post-operative pictures were taken, and patients were reassessed, evaluating aspects such as difference in skin pigmentation between the reconstructed site and adjacent areas, donor site morbidity, altered and depressed contour of the reconstructed site, constriction of the external auditory canal, and ear asymmetry.

### 2.1. Surgical Technique

Intraoperative antibiotic prophylaxis with amoxicillin was administered in all cases. Most procedures were performed under local anesthesia by injecting carbocaine and ropivacaine with epinephrine in the subcutaneous plane. In the case of the local flap, the donor area was also injected. We applied the same protocol even in the case of patients undergoing general anesthesia to prevent massive bleeding and post-operative pain. After surgery, patients were evaluated for early complications.

The surgical reconstructive procedures performed in this study included full thickness skin grafts and local flaps. The Antia-Buch flap was performed in the case of defects of the helix greater than 1.5 cm with cartilage involvement. This flap requires incisions to be placed along the helical sulcus inferiorly, superiorly, or in both directions. Incisions should avoid damaging the skin of the posterior aspect of the ear, as this ensures the vascular supply to the flaps. Further mobilization of the upper segment can be achieved through a V-Y advancement flap of the helix root, which allows the advancement of the upper flap to be extended, and, therefore, was used for larger defects. The preauricular flap is designed to exploit the skin laxity of the preauricular area and its design should not exceed a 4:1 length to width ratio. The flap can be harvested thinner in its distal aspect to match the thickness of the skin of the auricle, and thicker at the level of the pedicle to ensure blood supply to the tissues. It can reach the defect at the ear level directly or by creating a tunnel under healthy skin according to reconstructive requirements. In our study, it was used for antihelix, tragus, antitragus, and lobule defects. The retroauricular flap was used for the reconstruction of the marginal defects of the helix without cartilage involvement. The flap is designed on the posterior aspect of the ear and advanced to cover the defect. It provides an excellent skin match without the need for reduction in the size of the auricle. The “revolving door” flap was used to reconstruct the anterior auricular defects of the scapha and concha when the cartilage was removed. The surgical technique involves the rotation of the flap from the retroauricular to the preauricular surface on a vertical axis represented by the neurovascular subcutaneous peduncle at the level of the retroauricular sulcus. The lobular flaps were used for the reconstruction of the lower aspect of the ear (antihelix, tragus, and antitragus) when an excess of skin was found at the level of the lobule. This is usually possible in elderly patients.

### 2.2. Statistical Snalysis

Statistical analysis was performed with SPSS software (IBM Corp., Armonk, NY, USA). The relationships between patients’ demographics and complication rates were analyzed by chi-square tests. The statistical significance was defined as *p* < 0.05.

## 3. Results

From February 2018 to November 2020, a total of 49 patients underwent auricle reconstruction, but three of them were excluded from the study as they died during follow-up, due to circumstances not related to the cause of reconstruction. Among the 46 patients included in the study, 36 were males and 10 were females. The average patient age was 79.9 years (range: 61 to 94 years). Regarding additional patients’ demographics, 14 patients were smokers (30.4%), none of the patients were diabetic, and 16 patients were affected by cardiovascular disease and were on anticoagulants (34.8%). 

The most common cause for ear reconstruction was represented by basal cell carcinoma (20–43.5%), followed by squamous cell carcinoma (16–34.8%) and precancerous lesions (10–21.7%) (Table 1).

The standard excision limits for the primary lesions were ≥0.5 mm. Among patients affected by squamous cell carcinoma, 11 lesions were 2 cm or less in greatest dimension and 5 lesions were between 2 and 4 cm in greatest dimension. No patients showed lymph node involvement prior to tumor excision nor during follow-up.

The mean area of defect was 4.3 cm^2^. Regarding defect location, in 24 cases the helix was affected, in 14 cases the antihelix was involved, 4 cases required lobule reconstruction, in 2 cases the tumor affected the tragus and antitragus, and in 2 cases the concha was involved (Table 2).

The most commonly performed reconstructive procedures were Antia-Buch flap (30.4%), followed by wedge excision (26.1%), preauricular flap (13.0%), retroauricular flap (8.7%), lobular flap (8.7%), full thickness skin graft (8.7%), and “revolving door” flap (4.3%). In terms of post-operative complications, one patient (2.2%) developed a post-operative infection treated with antibiotics, and another patient (2.2%), who was treated with a skin graft and experienced partial graft loss, resolved with secondary healing not requiring additional procedures (Table 3). The relationship between complication rates and patients’ demographics, such as smoking status and use of anticoagulant medications, was tested and found to be not significant (*p* > 0.05). 

At a minimum follow-up time of 12 months (mean time from surgical procedure of 22 months), the characteristics related to the outcomes of the reconstruction were evaluated. In particular, a difference in skin pigmentation of the reconstructed site compared to the adjacent areas was reported in 10 cases (21.7%). For none of the cases in which a donor site was used for reconstruction, relevant issues were identified with the donor site itself. A depressed contour of the ear at the level of the reconstruction was highlighted in four (8.7%) cases. No cases of a constriction of the external auditory canal were recorded. In 11 cases (23.9%), however, a moderate ear asymmetry with the contralateral was found. Secondary surgeries were not performed in any cases, neither for enhancing cosmetic outcomes nor due to recurrence of disease (Table 4).

### Algorithm

**Figure 1:**
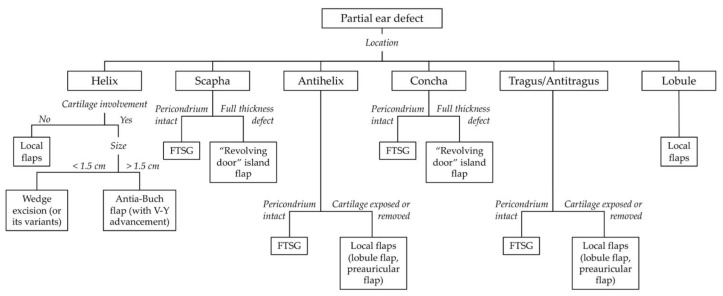
Reconstructive algorithm for partial defects of the ear (FTSG: Full thickness skin graft).

In this study, the defects were reconstructed according to location, size of defect, and tissue involved (Figure 1). In particular, according to location, defects were classified into affecting the helix, the scapha, the antihelix, the concha, the lobule, the tragus, and antitragus. With relation to tissue involvement, attention was paid to whether the cartilage was involved by the tumor and needed to be resected.

**Figure 2:**
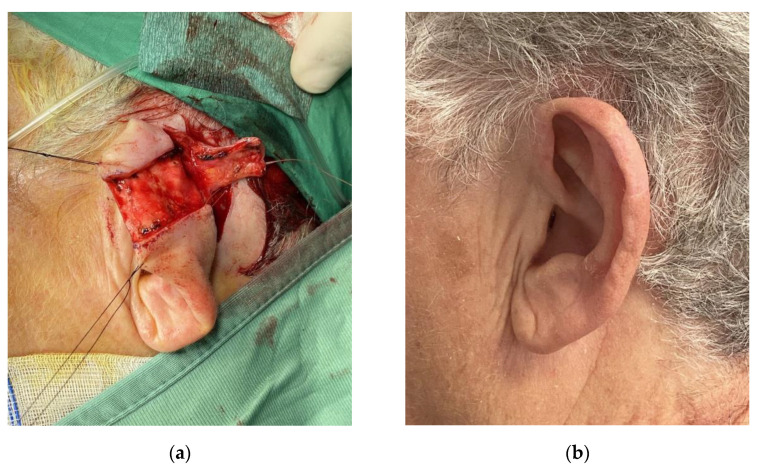
Retroauricular flap used to reconstruct a defect of the helical region without cartilage involvement. (**a**) Flap harvest from the posterior aspect of the auricle; (**b**) One-year follow-up.

In the upper aspect of the ear, it is appropriate to consider cartilage involvement when considering defects affecting the helical region. If it was possible to spare the cartilage, we performed a retroauricular advancement flap by lifting the flap from the retroauricular region and advancing it horizontally until full coverage of the defect was achieved (Figure 2). 

**Figure 3:**
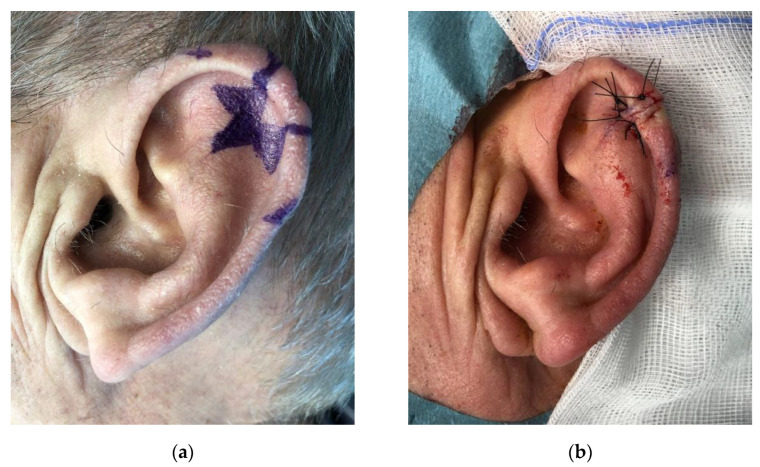
Star modification of wedge excision for a lesion of the helical region. (**a**) Preoperative markings; (**b**) Closure of the defect.

On the other hand, if the cartilage was involved, the size of the defect was considered. For lesions smaller than 1.5 cm, we performed a wedge-shaped excision of the lesion or its variants (star resection) to reduce the tension resulting from juxtaposing the edges of the defect (Figure 3). 

**Figure 4:**
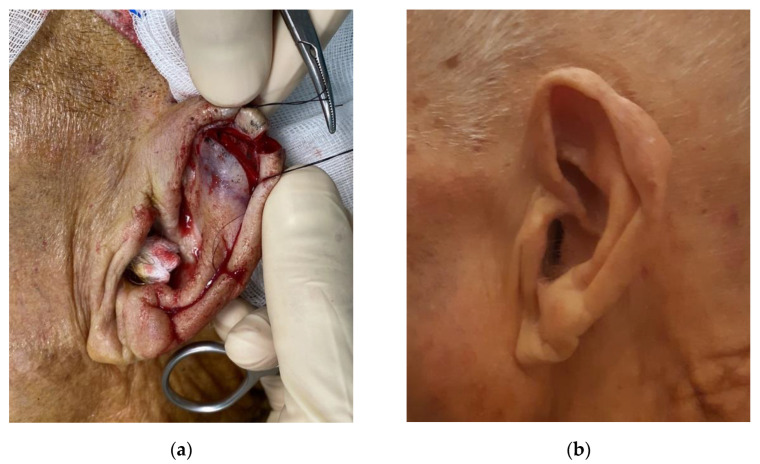
Antia-Buch flap. (**a**) Flap harvest; (**b**) One-year follow-up.

For defects larger than 1.5 cm, but less than 40% or the whole length of the auricle, we performed the Antia-Buch flap (Figure 4). 

**Figure 5:**
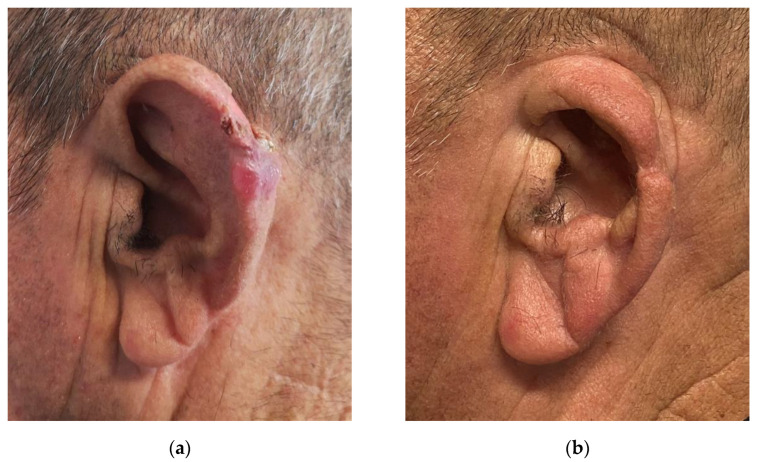
Modified Antia-Buch flap with V-Y advancement. (**a**) Preoperative view of the lesion; (**b**) One-year follow-up.

If needed, a V-Y advancement flap was performed at the helix root to obtain an additional advancement (Figure 5). 

**Figure 6:**
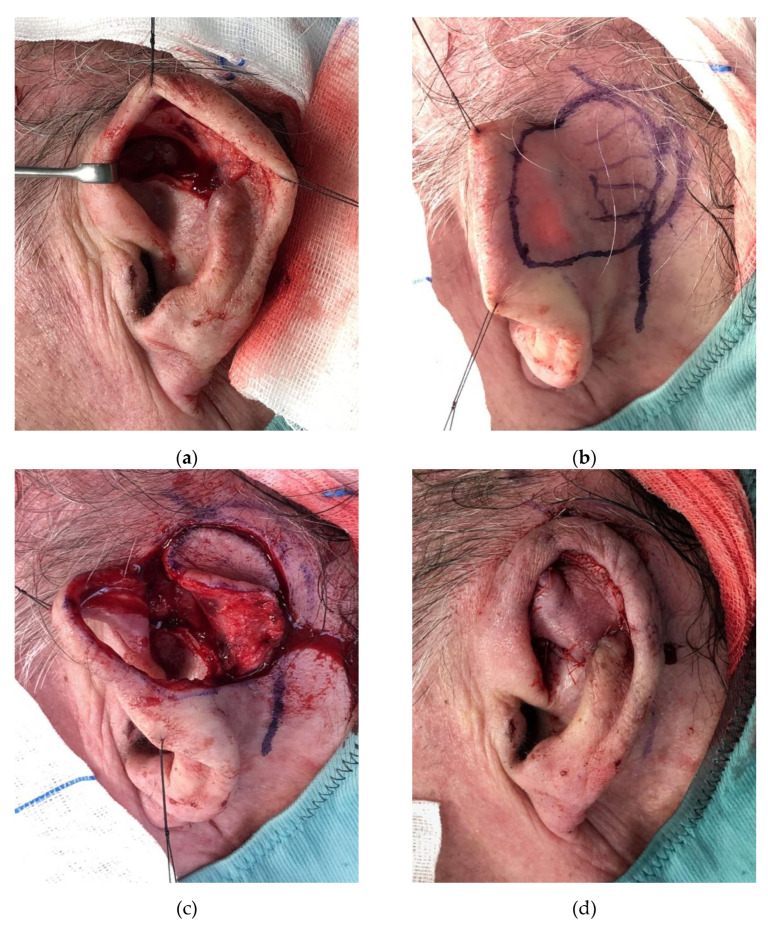
“Revolving door” island flap. (**a**) Preoperative view of the defect; (**b**) Preoperative markings; (**c**) Flap harvest; (**d**) Closure of the defect.

Still considering the upper aspect of the ear, if the scapha and antihelix region was involved but not the helix, and the perichondrium was intact, a full thickness skin graft was used to cover the defect. On the other hand, if the cartilage was removed, we performed a ”revolving door” island flap (Figure 6).

**Figure 7:**
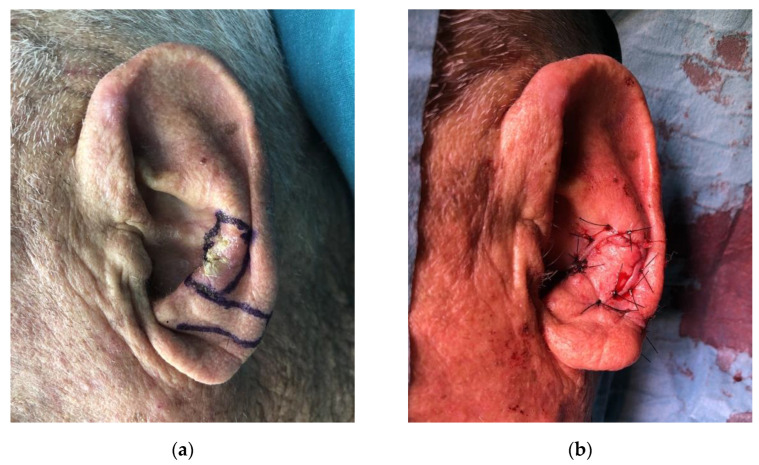
Local lobule flap. (**a**) Preoperative view of the lesion and markings; (**b**) Closure of the defect.

Regarding the middle aspect of the ear, the same considerations presented before were applied to the helix area. If the defect involved the antihelix but spared the ear margin, a local lobule flap was performed (Figure 7). 

**Figure 8:**
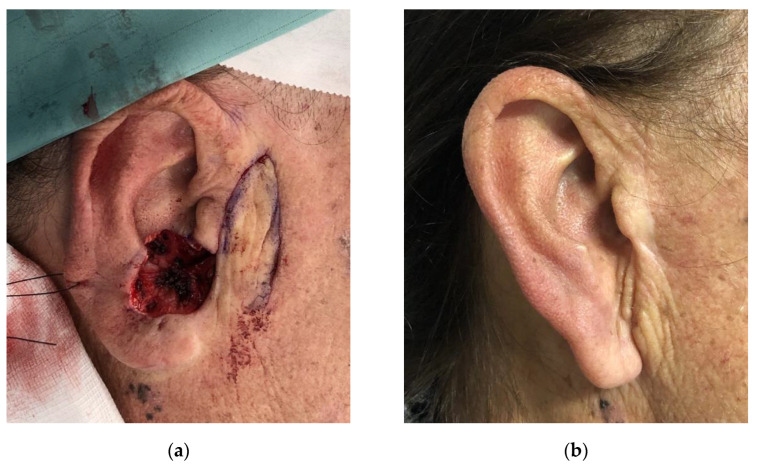
Preauricular flap. (**a**) Intraoperative view of the defect and flap harvesting; (**b**) One-year follow-up.

If the perichondrium was intact, the defects of the concha were treated with a full thickness skin graft. Otherwise, the ”revolving door” island flap was used. Preauricular flaps were used to reconstruct the defects of the tragus, antitragus, and the lobule (Figure 8).

## 4. Discussion

Recreating the complex anatomy of the auricle after cancer excision still represents a challenge, particularly for young plastic surgeons. A wide range of procedures have been described to approach this topic, but it is not always easy to determine which is the most suitable for any specific situation. 

We retrospectively evaluated 46 patients treated at our institution for partial defects of the auricle. The most common cause for ear reconstruction was represented by basal cell carcinoma followed by squamous cell carcinoma. Patients in which the defect was caused by excision of the precancerous lesions were also included, as the reconstructive procedure followed the same principles. Even though conservative treatment of precancerous lesions is usually performed, in our cases the excision was suggested after thorough assessment by a dermatologist due to characteristics of the lesion which could not exclude its malignant nature [4,5]. In this study, we presented the criteria used for the selection of the reconstructive procedure, and the results in terms of complication rate and quality of the reconstruction. Other studies have been published on the topic by different authors, however, we evaluated the characteristics of the reconstruction after a long term follow up of a minimum of 12 months (mean follow-up time of 22 months) [6,7]. 

As ear reconstruction after cancer excision is often performed in elderly patients (mean age of 79.9 in our study), it is advisable to keep in mind that a simple yet effective reconstructive procedure, with low risks of complication, is usually the best choice. Interestingly, Sanniec et al. found no statistically significant difference in complication rate in elderly patients compared with younger patients [7]. Considering other factors, such as smoke habit, comorbidities (diabetes and cardiovascular disease), and drug assumptions (anticoagulants), we did not find a statistically significant correlation between these factors and the risk of complications. In our case series, the complications found were partial graft loss and infection, with a total rate of 4.4%. These findings are in line with the current literature on the topic [7,8,9]. 

At a long-term follow-up, we evaluated the quality of reconstruction by analyzing the different factors, such as difference in skin pigmentation between the reconstructed site and adjacent areas, donor site sequelae, altered and depressed contour of the reconstructed site, constriction of the external auditory canal, and ear asymmetry. In terms of the difference in skin pigmentation, this was noticed in 10 cases (21.7%) which were reconstructed with full thickness skin grafts and preauricular flaps. Skin grafts represent a simple and reliable procedure when only the skin is resected and the underlying perichondrium is intact, but they usually determine color mismatch and a longer healing time [10]. They can be harvested by the preauricular and retroauricular area or the supraclavicular area [6,11]. When the defect also involves the perichondrium, the cartilage may be resected if it does not provide structural support, allowing grafting on the perichondrium of the posterior side, or small holes can be performed on the cartilage to allow graft survival [12]. Otherwise, a local flap is needed to provide coverage for the exposed cartilage. The preauricular flap, used in different cases, provided adequate coverage with a minimal donor site morbidity, but resulted in color and texture mismatch. Despite this, the preauricular flap represents a very versatile solution for defects of the anterior ear [13]. The recent study published by Frattaroli et al. comparing the use of preauricular flaps and full thickness skin grafts in auricle reconstruction found a statistically significant difference in patients’ satisfaction in favor of the use of preauricular flaps [14]. Donor site morbidity was not reported in any of the cases in which a donor site was used for reconstruction, supporting the fact that the periauricular area is a very suitable area for tissue harvesting. 

In four cases (8.7%), a depressed contour of the ear at the level of the reconstruction was noticed. It must be stated that these were cases reconstructed with the Antia-Buch flap for defects greater than 2.5 cm. According to a cadaveric study performed by Calhoun et al., the helical rim advancement flaps provide satisfactory closure of helical rim defects up to at least 2 cm, but also longer in some cases, preserving the normal anatomic appearance [15]. This is further supported by other authors, who used the Antia-Buch flap for larger defects with satisfactory results [16]. In order to obtain an additional mobilization of the upper flap, a V-Y advancement can be performed at the level of the helix root. In our cases, despite the depressed contour of the reconstructed ear, this technique allowed us to perform a one stage reconstruction in larger defects without major deformities. For this reason, it is worth considering this approach, especially for the elderly patients. With regard to smaller defects of the helical rim, we performed a wedge excision, but if it resulted in a lateral protrusion of the ear and cupping, we used the star modification, which includes the excision of two additional full thickness triangles superiorly and inferiorly [17]. 

In our study, no case of constriction of the external auditory canal was recorded. This is a complication which may affect the reconstruction of the defects involving the concha [18]. In our experience, if the perichondrium is intact, a full thickness skin graft of the concha can be performed. Otherwise, for composite defects of this area, the “revolving door” island flap represents an excellent option [19,20,21]. Dessy et al. compared the outcomes of the reconstruction of the defects of the concha with full thickness skin grafts and “revolving door” island flaps, and found that the latest showed statistically significant higher scores in terms of the overall outcome, and the color and texture match [22]. 

Regarding ear asymmetry, even though a mild asymmetry was noticed at the follow-up of 11 patients, this was not found to be an issue as the vertical height of the helix root was maintained, hence allowing the patients to wear glasses. As no secondary procedures were performed to enhance cosmetic outcomes, we believe that our tailored approach to partial defects of the ear was successful in addressing common issues in auricle reconstruction. The selection of a single stage procedure was enthusiastically accepted by the elderly patients and, in our opinion, represents a valuable solution, especially during the COVID-19 pandemic, as it reduces patient access to hospitals [23,24,25,26]. 

The aim of this paper is to present the authors’ experience with the tailored reconstruction of the complex structures of the face, which carry both an aesthetic and functional burden, as previously discussed in studies and reviews on the topic [27,28,29,30,31]. The current study presents some limitations, including the reduced size of the cohort of patients and the exclusion of patients affected by larger defects requiring a more complex reconstruction. The reason for this is that, in this study, we wanted to concentrate on partial defects, providing the young surgeon with a complete and easy to apply algorithm to solve most cases with a single procedure leading to satisfactory results at a long-term follow-up. Larger studies are in progress to confirm results and provide additional information on the topic.

## 5. Conclusions

The high anatomical complexity of the skin and cartilage of the auricle is an issue when addressing ear reconstruction. Since these procedures are often required in elderly patients, a simple yet effective and reliable approach is advisable. 

The goal of this exploratory pilot study is to propose a single-stage reconstructive approach for the elderly patients which is easy to apply, with low complication rates and good outcomes for both the patient and the surgeon. For this reason, we propose an algorithm to choose the most suitable technique for each specific partial defect based on size, location, and tissue involvement. 

## Figures and Tables

**Table 1:** Patients’ demographics.

Patients’ Demographics	*n* (%)
Patients	46
Mean age, years	79.9
Gender - Males - Females	36 (78.3) 10 (21.7)
Smoking habit - Smokers - Non-smokers	14 (30.4%) 32 (69.6%)
Anticoagulant assumption - Yes - No	16 (34.8%) 30 (65.2%)
Histology - Basal cell carcinoma - Squamous cell carcinoma - Precancerous lesion	20 (43.5%) 16 (34.8%) 10 (21.7%)

**Table 2:** Size and location of defect.

Variable	*n* (%)
Average area of defect, cm^2^	4.3
Location of defect - Helix - Antihelix - Lobule - Concha - Tragus and antitragus	24 (52.2%) 14 (30.4%) 4 (8.7%) 2 (4.3%) 2 (4.3%)

**Table 3:** Reconstructive procedures performed and complications.

Variable	*n* (%)
Reconstructive procedure - Antia-Buch flap - Wedge excision - Preauricular flap - Retroauricular flap - Lobular flap - Full thickness skin graft - “Revolving door” flap	14 (30.4%) 12 (26.1%) 6 (13.0%) 4 (8.7%) 4 (8.7%) 4 (8.7%) 2 (4.3%)
Complications, total - Infection - Partial graft loss	2 (4.4%) 1 (2.2%) 1 (2.2%)

**Table 4:** Outcomes at a minimum follow-up of one year.

Variable	*n* (%)
Difference in skin pigmentation	10 (21.7%)
Donor site morbidity	0 (0%)
Depressed ear contour	4 (8.7%)
Constriction of the external auditory canal	0 (0%)
Moderate ear asymmetry	11 (23.9%)
Secondary surgery	0 (0%)

## Data Availability

The data presented in this study are available on request from the corresponding author (A.B.). The data are not publicly available due to privacy restrictions.

## References

[1] Smith R.M., Byrne P.J. (2019). Reconstruction of the ear. Facial Plast. Surg. Clin. N. Am..

[2] Johnson T.M., Fader D.J. (1997). The staged retroauricular to auricular direct pedicle (interpolation) flap for helical ear reconstruction. J. Am. Acad. Dermatol..

[3] Butler C.E. (2002). Extended retroauricular advancement flap reconstruction of a full-thickness auricular defect including posteromedial and retroauricular skin. Ann. Plast. Surg..

[4] Steeb T., Wessely A., Petzold A., Schmitz L., Dirschka T., Berking C., Heppt M.V. (2021). How to assess the efficacy of interventions for actinic keratosis? A review with a focus on long-term results. J. Clin. Med..

[5] Cantisani C., Paolino G., Scarnò M., Didona D., Tallarico M., Moliterni E., Losco L., Cantoresi F., Mercuri S.R., Bottoniτ U. (2018). Sequential methyl-aminolevulinate daylight photodynamic therapy and diclofenac plus hyaluronic acid gel treatment for multiple actinic keratosis evaluation. Dermatol. Ther..

[6] Shonka D.C., Park S.S. (2009). Ear defects. Facial Plast. Surg. Clin. North Am..

[7] Sanniec K., Harirah M., Thornton J.F. (2019). Ear reconstruction after Mohs cancer excision: Lessons learned from 327 consecutive cases. Plast. Reconstr. Surg..

[8] Kolodzynski M.N., Kon M., Egger S., Breugem C.C. (2017). Mechanisms of ear trauma and reconstructive techniques in 105 consecutive patients. Eur. Arch. Otorhinolaryngol..

[9] Schonauer F., Vuppalapati G., Marlino S., Santorelli A., Canta L., Molea G. (2010). Versatility of the posterior auricular flap in partial ear reconstruction. Plast. Reconstr. Surg..

[10] Trufant J.W., Marzolf S., Leach B.C., Cook J. (2016). The utility of full-thickness skin grafts (FTSGs) for auricular reconstruction. J. Am. Acad. Dermatol..

[11] Brodland D.G. (2005). Auricular reconstruction. Dermatol. Clin..

[12] Calhoun K.H., Chase S.P. (2005). Reconstruction of the auricle. Facial Plast. Surg. Clin. N. Am..

[13] Sorkin A., Heller L., Landau G., Sherf M., Hartstein M.E., Hadad E. (2020). Inferiorly based preauricular flap for anterior ear reconstruction. Ann. Plast. Surg..

[14] Frattaroli J.M., Turriziani G., Torto F.L., Cavalieri E., Bruno E., Prà G.D., Ribuffo D. (2021). The use of preauricular skin flaps in the treatment of ear malignancy in elderly patients. J. Cosmet. Dermatol..

[15] Calhoun K.H., Slaughter D., Kassir R., Seikaly H., Hokanson J.A. (1996). Biomechanics of the helical rim advancement flap. Arch. Otolaryngol. Head Neck Surg..

[16] De Schipper H.J.P., van Rappard J.H.A., Dumont E.A.W.J. (2012). Modified Antia Buch repair for full-thickness middle auricular defect. Dermatol. Surg..

[17] Bumsted R.M., Ceilley R.I. (1980). Stellate excision of malignancies on the auricles. J. Dermatol. Surg. Oncol..

[18] Luong A., Roland P. (2005). Acquired external auditory canal stenosis: Assessment and management. Curr. Opin. Otolaryngol. Head Neck Surg..

[19] Talmi Y.P., Wolf M., Horowitz Z., Bedrin L., Kronenberg J. (2002). “Second look” at auricular reconstruction with a postauricular island flap: “Flip-flop flap”. Plast. Reconstr. Surg..

[20] Humphreys T.R., Goldberg L.H., Wiemer R.D. (1996). The postauricular (revolving door) island pedicle flap revisited. Dermatol. Surg..

[21] Golash A., Bera S., Kanoi A.V., Golash A. (2020). The revolving door flap: Revisiting an elegant but forgotten flap for ear defect reconstruction. Indian J. Plast. Surg..

[22] Dessy L.A., Figus A., Fioramonti P., Mazzocchi M., Scuderi N. (2010). Reconstruction of anterior auricular conchal defect after malignancy excision: Revolving-door flap versus full-thickness skin graft. J. Plast. Reconstr. Aesthetic Surg..

[23] Spadea T., Di Girolamo C., Landriscina T., Leoni O., Forni S., Colais P., Fanizza C., Allotta A., Onorati R., Gnavi R. (2021). Indirect impact of Covid-19 on hospital care pathways in Italy. Sci. Rep..

[24] Van der Molen D.R.M., Bargon C.A., Batenburg M.C.T., van Stam L.E., van Dam I.E., Baas I.O., Ernst M.F., Maarse W., Sier M., Schoenmaeckers E.J.P. (2021). The impact of the COVID-19 pandemic on perceived access to health care and preferences for health care provision in individuals (being) treated for breast cancer. Breast Cancer Res. Treat..

[25] Marcasciano M., Kaciulyte J., Mori F.L.R., Lo Torto F., Barellini L., Loreti A., Fanelli B., De Vita R., Redi U., Marcasciano F. (2020). Breast surgeons updating on the thresholds of COVID-19 era: Results of a multicenter collaborative study evaluating the role of online videos and multimedia sources on breast surgeons education and training. Eur. Rev. Med. Pharmacol. Sci..

[26] Mehrabian D., Liu I.Z., Pakhchanian H.H., Tarawneh O.H., Raiker R., Boyd C.J. (2021). Nationwide analysis of plastic and reconstructive procedural volume in the United States during the COVID-19 pandemic. J. Plast. Reconstr. Aesthetic Surg..

[27] Losco L., Aksoyler D., Chen S.H., Bolletta A., Velazquez-Mujica J., Di Taranto G., Torto F.L., Marcasciano M., Cigna E., Chen H.C. (2020). Pharyngoesophageal reconstruction with free jejunum or radial forearm flap as diversionary conduit: Functional outcomes of patients with persistent dysphagia and aspiration. Microsurgery.

[28] Lo Torto F., Losco L., Bernardini N., Greco M., Scuderi G., Ribuffo D. (2017). Surgical treatment with locoregional flaps for the eyelid: A review. BioMed Res. Int..

[29] Losco L., Bolletta A., Pierazzi D., Spadoni D., Cuomo R., Marcasciano M., Cavalieri E., Roxo A., Ciamarra P., Cantisani C. (2020). Reconstruction of the nose: Management of nasal cutaneous defects according to aesthetic subunit and defect size. A review. Medicina.

[30] Bulla A., Vielà C., Fiorot L., Bolletta A., Pancrazi E., Campus G.V. (2017). A new approach to upper eyelid reconstruction. Aesthetic Plast. Surg..

[31] Paolino G., Cardone M., Didona D., Moliterni E., Losco L., Corsetti P., Schipani G., Lopez T., Calvieri S., Bottoni U. (2020). Prognostic factors in head and neck melanoma according to facial aesthetic units. G. Ital. Dermatol. Venereol..

